# International Veterinary Epilepsy Task Force consensus proposal: medical treatment of canine epilepsy in Europe

**DOI:** 10.1186/s12917-015-0464-z

**Published:** 2015-08-28

**Authors:** Sofie F.M. Bhatti, Luisa De Risio, Karen Muñana, Jacques Penderis, Veronika M. Stein, Andrea Tipold, Mette Berendt, Robyn G. Farquhar, Andrea Fischer, Sam Long, Wolfgang Löscher, Paul J.J. Mandigers, Kaspar Matiasek, Akos Pakozdy, Edward E. Patterson, Simon Platt, Michael Podell, Heidrun Potschka, Clare Rusbridge, Holger A. Volk

**Affiliations:** Department of Small Animal Medicine and Clinical Biology, Faculty of Veterinary Medicine, Ghent University, Salisburylaan 133, Merelbeke, 9820 Belgium; Animal Health Trust, Lanwades Park, Kentford, Newmarket, CB8 7UU Suffolk, United Kingdom; Department of Clinical Sciences, College of Veterinary Medicine, North Carolina State University, 1052 William Moore Drive, Raleigh, NC 27607 USA; Vet Extra Neurology, Broadleys Veterinary Hospital, Craig Leith Road, Stirling, FK7 7LE Stirlingshire United Kingdom; Department of Small Animal Medicine and Surgery, University of Veterinary Medicine Hannover, Bünteweg 9, 30559 Hannover, Germany; Department of Veterinary and Clinical Sciences, Faculty of Health and Medical Sciences, University of Copenhagen, Frederiksberg C, Denmark; Fernside Veterinary Centre, 205 Shenley Road, Borehamwood, SG9 0TH Hertfordshire, United Kingdom; Clinical Veterinary Medicine, Ludwig-Maximillians-University, Veterinärstr. 13, 80539 Munich, Germany; University of Melbourne, 250 Princes Highway, Weibee, 3015 VIC Australia; Department of Pharmacology, Toxicology and Pharmacy, University of Veterinary Medicine Hannover, Bünteweg 17, 30559 Hannover, Germany; Department of Clinical Sciences of Companion Animals, Utrecht University, Yalelaan 108, 3583 CM Utrecht, The Netherlands; Section of Clinical & Comparative Neuropathology, Centre for Clinical Veterinary Medicine, Ludwig-Maximilians-University, Veterinärstr. 13, 80539 Munich, Germany; Clinical Unit of Internal Medicine Small Animals, University of Veterinary Medicine, Veterinärplatz 1, 1210 Vienna, Austria; University of Minnesota College of Veterinary Medicine, D426 Veterinary Medical Center, 1352 Boyd Avenue, St. Paul, MN 55108 USA; College of Veterinary Medicine, University of Georgia, 501 DW Brooks Drive, Athens, GA 30602 USA; Chicago Veterinary Neurology and Neurosurgery, 3123 N. Clybourn Avenue, Chicago, IL 60618 USA; Department of Pharmacology, Toxicology and Pharmacy, Ludwig-Maximillians-University, Königinstr. 16, 80539 Munich, Germany; Fitzpatrick Referrals, Halfway Lane, Eashing, Godalming, GU7 2QQ Surrey, United Kingdom; School of Veterinary Medicine, Faculty of Health & Medical Sciences, University of Surrey, Guildford, GU2 7TE Surrey, United Kingdom; Department of Clinical Science and Services, Royal Veterinary College, Hatfield, AL9 7TA Hertfordshire UK

**Keywords:** Dog, Epileptic seizure, Epilepsy, Treatment

## Abstract

In Europe, the number of antiepileptic drugs (AEDs) licensed for dogs has grown considerably over the last years. Nevertheless, the same questions remain, which include, 1) when to start treatment, 2) which drug is best used initially, 3) which adjunctive AED can be advised if treatment with the initial drug is unsatisfactory, and 4) when treatment changes should be considered. In this consensus proposal, an overview is given on the aim of AED treatment, when to start long-term treatment in canine epilepsy and which veterinary AEDs are currently in use for dogs. The consensus proposal for drug treatment protocols, 1) is based on current published evidence-based literature, 2) considers the current legal framework of the cascade regulation for the prescription of veterinary drugs in Europe, and 3) reflects the authors’ experience. With this paper it is aimed to provide a consensus for the management of canine idiopathic epilepsy. Furthermore, for the management of structural epilepsy AEDs are inevitable in addition to treating the underlying cause, if possible.

## Background

In Europe, the number of antiepileptic drugs (AEDs) licensed for dogs has grown considerably over the last years. Nevertheless, the same questions remain, which include, 1) when to start treatment, 2) which drug is best used initially, 3) which adjunctive AED can be advised if treatment with the initial drug is unsatisfactory, and 4) when treatment changes should be considered. In this consensus proposal, an overview is given on the aim of AED treatment, when to start long-term treatment in canine epilepsy and which veterinary AEDs are currently in use for dogs. The consensus proposal for drug treatment protocols, 1) is based on current published evidence-based literature [17], 2) considers the current legal framework of the cascade regulation for the prescription of veterinary drugs in Europe, and 3) reflects the authors’ experience. With this paper it is aimed to provide a consensus for the management of canine idiopathic epilepsy. Furthermore, for the management of structural epilepsy AEDs are inevitable in addition to treating the underlying cause, if possible.

At present, there is no doubt that the administration of AEDs is the mainstay of therapy. In fact, the term AED is rather inappropriate as the mode of action of most AEDs is to suppress epileptic seizures, not epileptogenesis or the pathophysiological mechanisms of epilepsy. Perhaps, in the future, the term anti-seizure drugs might be more applicable in veterinary neurology, a term that is increasingly used in human epilepsy. Additionally, it is known that epileptic seizure frequency appears to increase over time in a subpopulation of dogs with untreated idiopathic epilepsy, reflecting the need of AED treatment in these patients [63].

In our consensus proposal on classification and terminology we have defined idiopathic epilepsy as a disease in its own right, *per se*. A genetic origin of idiopathic epilepsy is supported by genetic testing (when available) and a genetic influence is supported by a high breed prevalence (>2 %), genealogical analysis and /or familial accumulation of epileptic individuals. However in the clinical setting idiopathic epilepsy remains most commonly a diagnosis of exclusion following diagnostic investigations for causes of reactive seizures and structural epilepsy.

### Aims of AED treatment

The ideal goal of AED therapy is to balance the ability to eliminate epileptic seizures with the quality of life of the patient. Seizure eradication is often not likely in dogs. More realistic goals are to decrease seizure frequency, duration, severity and the total number of epileptic seizures that occur over a short time span, with no or limited and acceptable AED adverse effects to maximize the dog’s and owner’s quality of life. Clinicians should approach treatment using the following paradigm [23, 76, 91, 92, 120]:

- **Decide when to start AED treatment**
- **Choose the most appropriate AED and dosage**
- **Know if and when to monitor serum AED concentrations and adjust treatment accordingly**
- **Know when to add or change to a different AED**
- **Promote pet owner compliance**

### When to recommend maintenance AED treatment?

Definitive, evidence-based data on when to start AED therapy in dogs based on seizure frequency and type is lacking. As such, extrapolation from human medicine may be possible to provide treatment guidelines. Clinicians should consider the general health of the patient, as well as the owner’s lifestyle, financial limitations, and comfort with the proposed therapeutic regimen. Individualized therapy is paramount for choosing a treatment plan. As a general rule, the authors recommend initiation of long-term treatment in dogs with idiopathic epilepsy when any one of the following criteria is present:

- **Interictal period of ≤ 6 months** (i.e. 2 or more epileptic seizures within a 6 month period)
- **Status epilepticus or cluster seizures**
- **The postictal signs are considered especially severe** (e.g. aggression, blindness) **or last longer than 24 hours**
- **The epileptic seizure frequency and/or duration is increasing and/or seizure severity is deteriorating over 3 interictal periods**

In humans, the decision regarding when to recommend AED treatment is based on a number of risk factors (e.g. risk of recurrence, seizure type, tolerability, adverse effects) [42, 115]. In people, clear proof exists that there is no benefit initiating AED treatment after a single unprovoked seizure [42], but there is evidence to support starting treatment after the second seizure [43, 108]. In dogs, long-term seizure management is thought to be most successful when appropriate AED therapy is started early in the course of the disease, especially in dogs with a high seizure density and in dog breeds known to suffer from a severe form of epilepsy [12−14]. A total number of ≥ 10 seizures during the first 6 months of the disease appeared to be correlated with a poor outcome in Australian Shepherds with idiopathic epilepsy [132]. Furthermore, recent evidence exists that seizure density is a crucial risk factor, experiencing cluster seizures, and being male is associated with poor AED response [84].

A strong correlation exists in epileptic people between a high seizure frequency prior to AED treatment and poor AED response [16, 34, 59]. Historically, this has been attributed to kindling, in which seizure activity leads to intensification of subsequent seizures [117]. However, there is little clinical evidence that kindling plays a role in either dogs [54] or humans [111] with recurrent seizures. In humans, a multifactorial pathogenesis is suggested [14, 52]. Recent epidemiologic data suggest that there are differences in the intrinsic severity of epilepsy among individuals, and these differences influence a patient’s response to medication and long-term outcome. Additionally, evidence for seizure-associated alterations that affect the pharmacodynamics and pharmacokinetics of AEDs have been suggested [99]. Breed-related differences in epilepsy severity have been described in dogs, with a moderate to severe clinical course reported in Australian Shepherds [132], Border Collies [49, 84], Italian Spinoni [24], German Shepherds and Staffordshire Bull Terriers [84], whereas a less severe form of the disease has been described in a different cohort of Collies (mainly rough coated) [77], Labrador Retrievers [7] and Belgian Shepherds [45]. Consequently, genetics may affect the success of treatment and may explain why some breeds are more predisposed to drug resistant epilepsy [3, 77].

### Choice of AED therapy

There are no evidence-based guidelines regarding the choice of AEDs in dogs. When choosing an AED for the management of epilepsy in dogs several factors need to be taken into account (AED-specific factors (e.g. regulatory aspects, safety, tolerability, adverse effects, drug interactions, frequency of administration), dog-related factors (e.g. seizure type, frequency and aetiology, underlying pathologies such as kidney/hepatic/gastrointestinal problems) and owner-related factors (e.g. lifestyle, financial circumstances)) [23]. In the end, however, AED choice is often determined on a case-by-case basis.

Until recently, primary treatment options for dogs with epilepsy have focused mainly on phenobarbital (PB) and potassium bromide (KBr) due to their long standing history, widespread availability, and low cost. While both AEDs are still widely used in veterinary practice, several newer AEDs approved for use in people are also being used for the management of canine idiopathic epilepsy mainly as add-on treatment. Moreover, since early 2013, imepitoin has been introduced in most European countries for the management of recurrent single generalized epileptic seizures in dogs with idiopathic epilepsy.

Several AEDs of the older generation approved for humans have been shown to be unsuitable for use in dogs as most have an elimination half-life that is too short to allow convenient dosing by owners, these include phenytoin, carbamazepine, valproic acid, and ethosuximide [119]. Some are even toxic in dogs such as lamotrigine (the metabolite is cardiotoxic) [26, 136] and vigabatrin (associated with neurotoxicity and haemolytic anemia) [113, 131, 138].

Since the 1990s, new AEDs with improved tolerability, fewer side effects and reduced drug interaction potential have been approved for the management of epilepsy in humans. Many of these novel drugs appear to be relatively safe in dogs, these include levetiracetam, zonisamide, felbamate, topiramate, gabapentin, and pregabalin. Pharmacokinetic studies on lacosamide [68] and rufinamide [137] support the potential use of these drugs in dogs, but they have not been evaluated in the clinical setting. Although these newer drugs have gained considerable popularity in the management of canine epilepsy, scientific data on their safety and efficacy are very limited and cost is often prohibitive.

### Phenobarbital

#### Efficacy

PB has the longest history of chronic use of all AEDs in veterinary medicine. After decades of use, it has been approved in 2009 for the prevention of seizures caused by generalized epilepsy in dogs. PB has a favourable pharmacokinetic profile and is relatively safe [2, 87, 97]. PB seems to be effective in decreasing seizure frequency in approximately 60−93 % of dogs with idiopathic epilepsy when plasma concentrations are maintained within the therapeutic range of 25−35 mg/l [10, 31, 74, 105]. According to Charalambous et al. (2014) [17], there is overall good evidence for recommending the use of PB as a monotherapy AED in dogs with idiopathic epilepsy. Moreover, the superior efficacy of PB was demonstrated in a randomized clinical trial comparing PB to bromide (Br) as first-line AED in dogs, in which 85 % of dogs administered PB became seizure-free for 6 months compared with 52 % of dogs administered Br [10]. This study demonstrated a higher efficacy of PB compared to Br as a monotherapy, providing better seizure control and showing fewer side effects.

### Pharmacokinetics

PB is rapidly (within 2h) absorbed after oral administration in dogs, with a reported bioavailability of approximately 90 % [2, 87]. Peak serum concentrations are achieved approximately 4−8h after oral administration in dogs [2, 97]. The initial elimination half-life in normal dogs has been reported to range from 37−73h after multiple oral dosing [96]. Plasma protein binding is approximately 45 % in dogs [36]. PB crosses the placenta and can be teratogenic.

PB is metabolized primarily by hepatic microsomal enzymes and approximately 25 % is excreted unchanged in the urine. There is individual variability in PB absorption, excretion and elimination half-life [2, 87, 97]. In dogs, PB is a potent inducer of cytochrome P450 enzyme activity in the liver [48], and this significantly increases hepatic production of reactive oxygen species, thus increasing the risk of hepatic injury [107]. Therefore PB is contraindicated in dogs with hepatic dysfunction. The induction of cytochrome P450 activity in the liver can lead to autoinduction or accelerated clearance of itself over time, also known as metabolic tolerance, as well as endogenous compounds (such as thyroid hormones) [40, 48]. As a result, with chronic PB administration in dogs, its total body clearance increases and elimination half-life decreases progressively which stabilizes between 30−45 days after starting therapy [97]. This can result in reduction of PB serum concentrations and therapeutic failure and therefore, monitoring of serum PB concentrations is very important for dose modulation over time.

A parenteral form of PB is available for intramuscular (IM) or intravenous (IV) administration. Different PB formulations are available in different countries, it should be emphasized, however, that IM formulations cannot be used IV and *vice versa*. Parenteral administration of PB is useful for administering maintenance therapy in hospitalized patients that are unable to take oral medication. The pharmacokinetics of IM PB have not been explored in dogs, however, studies in humans have shown a similar absorption after IM administration compared to oral administration [135]. The elimination half-life in dogs after a single IV dose is approximately 93h [87].

### Pharmacokinetic interactions

In dogs, chronic PB administration can affect the disposition of other co-administered medications which are metabolized by cytochrome P450 subfamilies and/or bound to plasma proteins [48]. PB can alter the pharmacokinetics and as a consequence may decrease the therapeutic effect of other AEDs (levetiracetam, zonisamide, and benzodiazepines) as well as corticosteroids, cyclosporine, metronidazole, voriconazole, digoxin, digitoxin, phenylbutazone and some anaesthetics (e.g. thiopental) [23, 33, 72, 82, 130]. As diazepam is used as first-line medicine for emergency use (e.g. status epilepticus) in practice it should be emphasized to double the IV or rectal dose of diazepam in dogs treated chronically with PB [130]. Concurrent administration of PB and medications that inhibit hepatic microsomal cytochrome P450 enzymes such as cimetidine, omeprazole, lansoprazole, chloramphenicol, trimethoprim, fluoroquinolones, tetracyclines, ketoconazole, fluconazole, itraconazole, fluoxetine, felbamate and topiramate may inhibit PB metabolism, increase serum concentration and can result in toxicity [10].

### Common adverse effects

Most of the adverse effects due to PB are dose dependent, occur early after treatment initiation or dose increase and generally disappear or decrease in the subsequent weeks due to development of pharmacokinetic and pharmacodynamic tolerance [35, 121] (Table 1). The adverse effects include sedation, ataxia, polyphagia, polydipsia and polyuria. For an in-depth review on the adverse effects of PB, the reader is referred to comprehensive book chapters [23, 32, 91].

**Table 1:** Most common reported adverse effects seen in dogs treated with PB, imepitoin and KBr (rarely reported and/or idiosyncratic adverse effects are indicated in grey

AED	Adverse effects in dogs
PB	Sedation
	Ataxia
	Polyphagia
	Polydipsia/polyuria
	
	
	
	
	
	
Imepitoin	Polyphagia (often transient)
	
KBr	Sedation
	Ataxia and pelvic limb weakness
	Polydipsia/polyuria
	Polyphagia
	Nausea, vomiting and/or diarrhea
	
	
	
	

### Idiosyncratic adverse effects

These effects occur uncommonly in dogs and include hepatotoxicity [13, 22, 39, 75], haematologic abnormalities (anaemia, and/or thrombocytopenia, and/or neutropenia) [51, 56]), superficial necrolytic dermatitis [66], potential risk for pancreatitis [38, 46], dyskinesia [58], anxiety [58], and hypoalbuminaemia [41] (Table 1). Most of these idiosyncratic reactions are potentially reversible with discontinuation of PB. For an in-depth review on the idiosyncratic adverse effects of PB the reader is referred to comprehensive book chapters [23, 32, 91].

### Laboratory changes

Laboratory changes related to chronic PB administration in dogs include elevation in serum liver enzyme activities [39, 41, 75], cholesterol and triglyceride concentrations [41]. Alterations in some endocrine function testing may occur (thyroid and adrenal function, pituitary-adrenal axis) [21, 41, 128]. For an in-depth review on these laboratory changes the reader is referred to comprehensive book chapters [23, 32, 91].

### Dose and monitoring (Fig. 1)

**Fig. 1:**
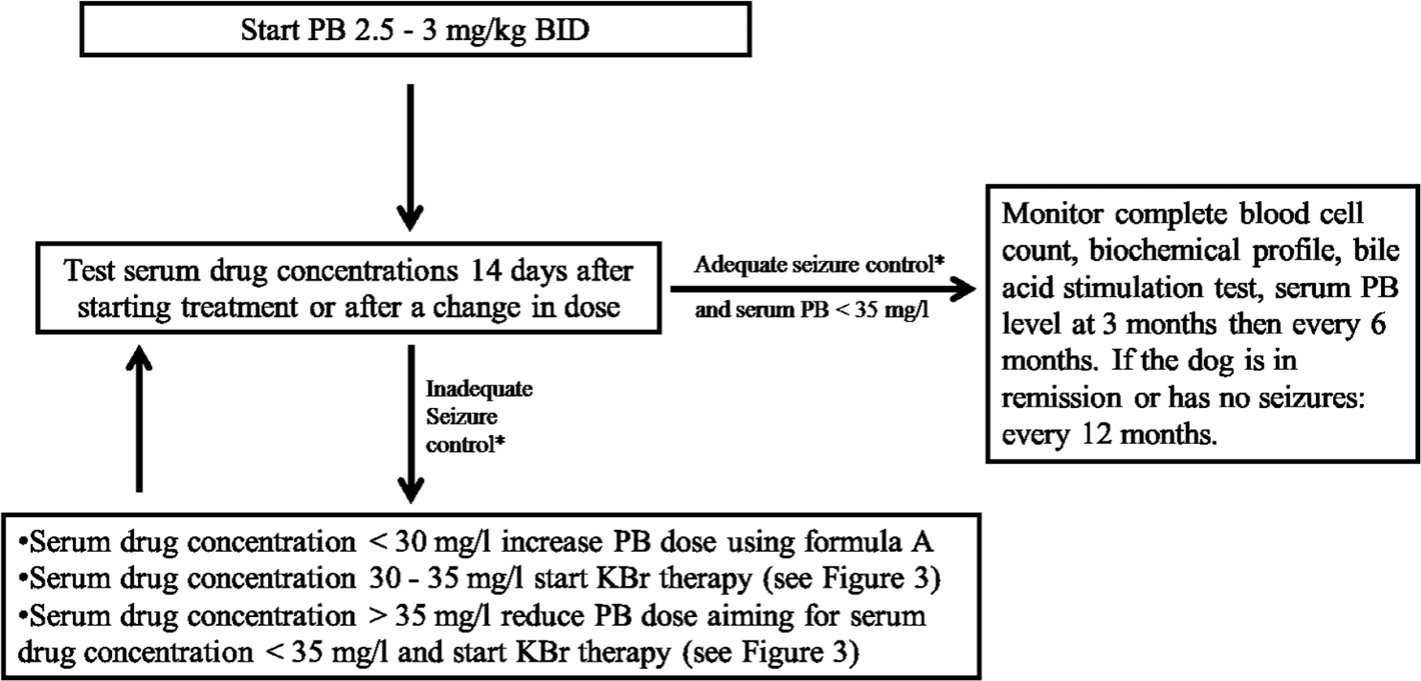
PB treatment flow diagram for decision making during seizure management in an otherwise healthy dog. The authors advise to start with PB (and add KBr if inadequate seizure control after optimal use of PB (Fig. 3)): in dogs with idiopathic epilepsy experiencing recurrent single generalised epileptic seizures; in dogs with idiopathic epilepsy experiencing cluster seizures or status epilepticus; in dogs with other epilepsy types. *Criteria for (in)adequate seizure control with regard to efficacy and tolerability (see Consensus proposal: Outcome of therapeutic interventions in canine and feline epilepsy [94]). 1. Treatment efficacious: a: Achievement of complete treatment success (i.e. seizure freedom or extension of the interseizure interval to three times the longest pretreatment interseizure interval and for a minimum of three months (ideally > 1 year); b: Achievement of partial treatment success (i.e. a reduction in seizure frequency including information on seizure incidence (usually at least 50 % or more reduction defines a drug responder), a reduction in seizure severity, or a reduction in frequency of seizure clusters and/or status epilepticus). 2. Treatment not tolerated i.e. appearance of severe adverse effects necessitating discontinuation of the AED

The recommended oral starting dose of PB in dogs is 2.5−3 mg/kg BID. Subsequently, the oral dosage is tailored to the individual patient based on seizure control, adverse effects and serum concentration monitoring.

Because of considerable variability in the pharmacokinetics of PB among individuals, the serum concentration should be measured 14 days after starting therapy (baseline concentration for future adjustments) or after a change in dose. To evaluate the effect of metabolic tolerance, a second PB serum concentration can be measured 6 weeks after initiation of therapy. Recommendations on optimal timing of blood collection for serum PB concentration monitoring in dogs vary among studies [23]. Generally, serum concentrations can be checked at any time in the dosing cycle as the change in PB concentrations through a daily dosing interval is not therapeutically relevant once steady-state has been achieved [62, 70]. However, in dogs receiving a dose of 5 mg/kg BID or higher, trough concentrations were significantly lower than non-trough concentrations and serum PB concentration monitoring at the same time post-drug dosing was recommended, in order to allow accurate comparison of results in these dogs [70]. Another study recommended performing serum PB concentration monitoring on a trough sample as a significant difference between peak and trough PB concentration was identified in individual dogs [10]. The therapeutic range of PB in serum is 15 mg/l to 40 mg/l in dogs. However, it is the authors’ opinion that in the majority of dogs a serum PB concentration between 25−30 mg/l is required for optimal seizure control. Serum concentrations of more than 35 mg/l are associated with an increased risk of hepatotoxicity and should be avoided [22, 75]. In case of inadequate seizure control, serum PB concentrations must be used to guide increases in drug dose. Dose adjustments can be calculated according to the following formula (Formula A):

$$ \mathrm{New}\ \mathrm{PB}\ \mathrm{total}\ \mathrm{daily}\ \mathrm{dosage}\ \mathrm{in}\ \mathrm{mg} = \left(\mathrm{desired}\ \mathrm{serum}\ \mathrm{PB}\ \mathrm{concentration}/\mathrm{actual}\ \mathrm{serum}\ \mathrm{PB}\ \mathrm{concentration}\right) \times \mathrm{actual}\ \mathrm{PB}\ \mathrm{total}\ \mathrm{daily}\ \mathrm{dosage}\ \mathrm{in}\ \mathrm{mg} $$

A dog with adequate seizure control, but serum drug concentrations below the reported therapeutic range, does not require alteration of the drug dose, as this serum concentration may be sufficient for that individual. Generally, the desired serum AED concentration for individual patients should be the lowest possible concentration associated with >50 % reduction in seizure frequency or seizure-freedom and absence of intolerable adverse effects [23].

In animals with cluster seizures, status epilepticus or high seizure frequency, PB can be administered at a loading dose of 15−20 mg/kg IV, IM or PO divided in multiple doses of 3−5 mg/kg over 24−48h to obtain a therapeutic brain concentration quickly and then sustain it [10]. Serum PB concentrations can be measured 1−3 days after loading. Some authors load as soon as possible (over 40 to 60 min) and start with a loading dose of 10 to 12 mg/kg IV followed by two further boluses of 4 to 6 mg/kg 20 min apart.

Complete blood cell count, biochemical profile (including cholesterol and triglycerides), and bile acid stimulation test should be performed before starting PB treatment and periodically at 3 months and then every 6 months during treatment. In case of adequate seizure control, serum PB concentrations should be monitored every 6 months. If the dog is in remission or has no seizures, a periodical control every 12 months is advised.

### Imepitoin

#### Efficacy

**Fig. 2:**
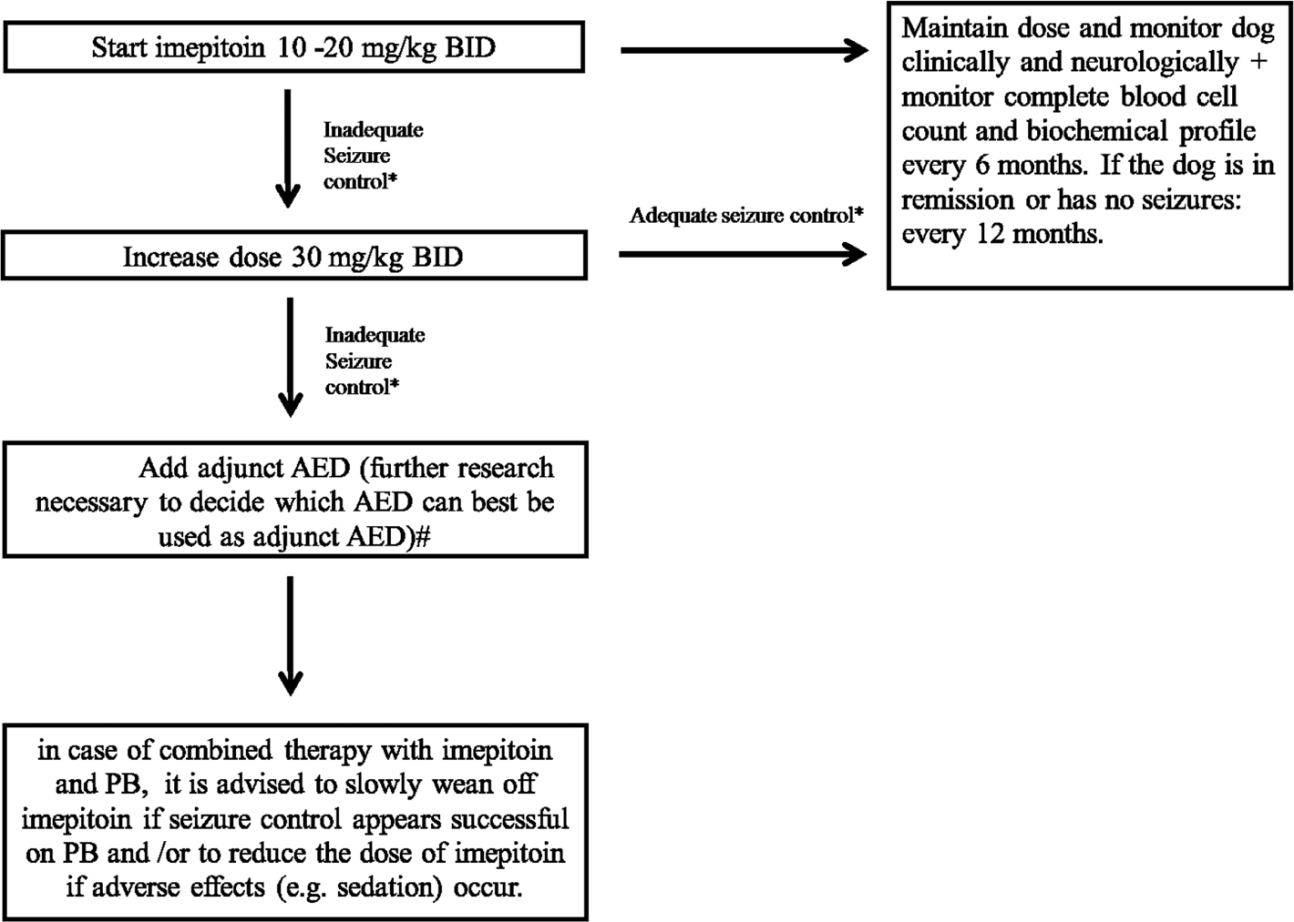
Imepitoin treatment flow diagram for decision making during seizure management in an otherwise healthy dog. The authors advise to start with imepitoin in dogs with idiopathic epilepsy experiencing recurrent single generalised epileptic seizures. *Criteria for (in)adequate seizure control with regard to efficacy and tolerability (see Consensus proposal: Outcome of therapeutic interventions in canine and feline epilepsy [94]). 1. Treatment efficacious: a: Achievement of complete treatment success (i.e. seizure freedom or extension of the interseizure interval to three times the longest pretreatment interseizure interval and for a minimum of three months (ideally > 1 year), b: Achievement of partial treatment success (i.e. a reduction in seizure frequency including information on seizure incidence (usually at least 50 % or more reduction defines a drug responder), a reduction in seizure severity, or a reduction in frequency of seizure clusters and/or status epilepticus). 2. Treatment not tolerated i.e. appearance of severe adverse effects necessitating discontinuation of the AED. ^#^Currently there are no data available on which AED should be added to imepitoin in case of inadequate seizure control. At this moment, the authors recommend the use of PB as adjunct AED in dogs receiving the maximum dose of imepitoin and experiencing poor seizure control

Imepitoin was initially developed as a new AED for humans, but, the more favourable pharmacokinetic profile of imepitoin in dogs versus humans led to the decision to develop imepitoin for the treatment of canine idiopathic epilepsy [102]. Based on randomized controlled trials that demonstrated antiepileptic efficacy, high tolerability and safety in epileptic dogs, the drug was approved in 2013 for this indication in Europe [64, 98, 122]. It has been recommended to use imepitoin in dogs with idiopathic epilepsy experiencing recurrent single generalized epileptic seizures, however, its efficacy has not yet been demonstrated in dogs with cluster seizures or status epilepticus [30]. In a recent randomized controlled study [122], the efficacy of imepitoin was compared with PB in 226 client-owned dogs. The administration of imepitoin twice daily in incremental doses of 10, 20 or 30 mg/kg demonstrated that the majority of dogs with idiopathic epilepsy were managed successfully with imepitoin without significant difference to the efficacy of PB. The frequency of adverse events (e.g. sedation, polydipsia, polyphagia) was significantly higher in the PB group [122]. In a study by Rieck et al. (2006) [98], dogs with chronic epilepsy not responding to PB or primidone received imepitoin (in its initial formulation) or KBr as adjunct AED and the seizure frequency improved to a similar degree in both groups. According to Charalambous et al. (2014) [17], there is good evidence for recommending the use of imepitoin as monotherapy in dogs with recurrent single generalized epileptic seizures, but insufficient evidence for use as adjunct AED. At present, scientific data and evidence-based guidelines on which AED can best be combined with imepitoin are lacking, and further research is needed. Nevertheless, at this moment, the authors recommend the use of PB as adjunct AED in dogs receiving the maximum dose of imepitoin and experiencing poor seizure control. According to the authors, in case of combined therapy with imepitoin and PB, it is advised to slowly wean off imepitoin over several months if seizure control appears successful on PB and/or to reduce the dose of imepitoin if adverse effects (e.g. sedation) occur (Fig. 2).

### Pharmacokinetics

Following oral administration of imepitoin at a dose of 30 mg/kg in healthy Beagle dogs, high plasma levels were observed within 30 min, but maximal plasma levels were only reached after 2−3h following a prolonged absorption time [101]. The elimination half-life was found to be short; approximately 1.5 to 2h. However, in another study in Beagle dogs, a longer half-life (~6 h) was found after higher doses of imepitoin, and accumulation of plasma levels was seen during chronic BID treatment [64]. Also, it has to be considered that Beagle dogs eliminate AEDs more rapidly than other dog strains [122]. Despite the short half-life in healthy Beagle dogs, this pharmacokinetic profile is reported as adequate to maintain therapeutically active concentrations with twice daily dosing in dogs [64, 122]. Imepitoin is extensively metabolized in the liver prior to elimination. In dogs, imepitoin is mainly excreted via the faecal route rather than the urinary route. Neither reduced kidney function nor impaired liver function is likely to greatly influence the pharmacokinetics of imepitoin [122].

### Pharmacokinetic interactions and adverse reactions

There is no information on pharmacokinetic interactions between imepitoin and other medications. Although, imepitoin is a low affinity partial agonist for the benzodiazepine binding site of the GABA_A_ receptor it has not prevented the pharmacological activity of full benzodiazepine agonists such as diazepam in the clinical setting (e.g. in dogs with status epilepticus) [122]. Consequently, because the affinity of diazepam for the GABA_A_ receptor is much higher than imepitoin, care should be taken in the emergency setting [122]. Therefore, dogs with idiopathic epilepsy treated with imepitoin and presented in status epilepticus might require, in addition to diazepam, an additional AED parenterally (e.g. PB, levetiracetam).

Mild and most commonly transient adverse reactions (Table 1) have been reported in dogs administered 10−30 mg/kg BID of imepitoin in its initial formulation; polyphagia at the beginning of the treatment, hyperactivity, polyuria, polydipsia, somnolence, hypersalivation, emesis, ataxia, lethargy, diarrhoea, prolapsed nictitating membranes, decreased vision and sensitivity to sound [64, 98].

As part of the development of imepitoin for the treatment of canine epilepsy, a target animal safety study in dogs was conducted [96]. Under laboratory conditions, healthy Beagle dogs were exposed to high doses (up to 150 mg/kg q12h) of imepitoin for 6 months. Clinical signs of toxicity were mild and infrequent and they were mostly CNS (depression, transient ataxia) or gastrointestinal system (vomiting, body weight loss, salivation) related. These clinical signs were not life-threatening and generally resolved within 24h if symptomatic treatment was given. These data indicate that imepitoin is a safe AED and is well tolerated up to high doses in dogs treated twice daily [96]. However, the safety of imepitoin has not been evaluated in dogs weighing less than 5 kg or in dogs with safety concerns such as renal, liver, cardiac, gastrointestinal or other disease. No idiosyncratic reactions have been demonstrated so far. The routinely measured liver enzymes’ activity do not appear to be induced by imepitoin [96]. Compared with the traditional benzodiazepines, such as diazepam, which acts as full agonists at the benzodiazepine site of the GABA_A_ receptor, partial agonists such as imepitoin show less sedative adverse effects and are not associated with tolerance and dependence during long-term administration in animal models [122]. Also in epileptic dogs, tolerance did not develop and no withdrawal signs were observed after treatment discontinuation [64].

### Dose and monitoring (Fig. 2)

The oral dose range of imepitoin is 10−30 mg/kg BID. The recommended oral starting dose of imepitoin is 10−20 mg/kg BID. If seizure control is not satisfactory after at least 1 week of treatment at this dose and the medication is well tolerated, the dose can be increased up to a maximum of 30 mg/kg BID. Reference range of plasma or serum imepitoin concentrations is unknown and there are no therapeutic monitoring recommendations for imepitoin from the manufacturer. Pharmacokinetic studies in dogs suggest variability in plasma imepitoin concentrations among individuals and sampling times. However, no correlation between plasma imepitoin concentration and seizure frequency reduction was identified [64] therefore and because of its wide therapeutic index, serum imepitoin monitoring is not needed.

The authors recommend a complete blood cell count and biochemical profile before starting imepitoin treatment and periodically every 6 months during treatment. If the dog is in remission or has no seizures, a periodical control every 12 months is advised.

### Bromide

#### Efficacy

Br is usually administered as the potassium salt (KBr). The sodium salt form (NaBr) contains more Br per gram of compound, therefore, the dose should be approximately 15 % less than that calculated for KBr [124]. In most EU countries, KBr is approved only for add-on treatment in dogs with epilepsy drug-resistant to first-line AED therapy. PB and KBr have a synergistic effect and add-on treatment with KBr in epileptic dogs improves seizure control in dogs that are poorly controlled with PB alone [46, 93, 126]. A recent study showed that KBr was less efficacious and tolerable than PB as first-line drug [10]. According to Charalambous et al. (2014) [17] there is fair level of evidence for recommending the use of KBr as a monotherapy, but less as adjunct AED.

### Pharmacokinetics

The bioavailability of Br after oral administration in normal dogs is approximately 46 %. The elimination half-life is long and ranges from 25−46 days in dogs, consequently, it can take several months (approximately 3 months) before steady-state concentrations after treatment initiation at maintenance dose are reached [46, 67, 90, 125]. KBr is unbound to plasma proteins and can diffuse freely across cellular membranes. KBr is not metabolised in the liver and is therefore a good alternative in dogs with hepatic dysfunction. KBr is excreted unchanged in the urine and undergoes tubular reabsorption in competition with chloride. Therefore, dietary factors affecting chloride levels can alter serum KBr concentrations [123]. High (low) dietary chloride concentrations increase (decrease) the excretion of KBr and shorten (prolong) its half-life. Dogs administered KBr should be maintained on a constant diet (and chloride intake) to prevent fluctuations in serum KBr concentrations, which could result in therapeutic failure or toxicity. If dietary changes are necessary they should be made gradually (over at least 5 days) and serum concentrations of KBr should be monitored following dietary changes, especially if the dog becomes sedated or has unexpected seizures. On biochemistry profiles serum chloride concentrations are often falsely elevated (“pseudohyperchloraemia”) because the assays cannot distinguish between chloride and Br ions [123].

### Pharmacokinetic interactions and adverse effects

Pharmacokinetic interactions of KBr are limited as KBr is not metabolized or protein-bound. The main interactions are associated with alterations in the renal excretion of KBr. As already mentioned, the rate of elimination of KBr varies proportionally and inversely to chloride intake. Loop diuretics such as furosemide may enhance KBr elimination by blocking KBr reabsorption through renal tubular chloride channels. KBr should be avoided in dogs with renal dysfunction to prevent toxicity secondary to reduced renal elimination [80].

Common, dose-dependent adverse effects of KBr in dogs include sedation, ataxia and pelvic limb weakness, polydipsia/polyuria, and polyphagia with weight gain [4, 25, 46, 124] (Table 1). These effects occur in the initial weeks of treatment and may be magnified by concurrent PB administration. These adverse effects subside (partly or completely), once KBr steady-state concentrations are reached [125]. Gastrointestinal irritation and clinical signs can be prevented or minimized by administering Br with food and dividing the daily dose into 2 or more doses [4].

Uncommon idiosyncratic reactions of KBr in dogs include personality changes (aggressive behaviour, irritability, hyperactivity), persistent cough, increased risk of pancreatitis and megaoesofagus [4, 46, 67, 106] (Table 1). Kbr may cause skin problems (bromoderma) in humans [106], but no reports exist currently in dogs. For an in-depth review on the adverse effects of Br the reader is referred to comprehensive book chapters [23, 32, 91].

### Dose and monitoring (Fig. 3)

**Fig. 3:**
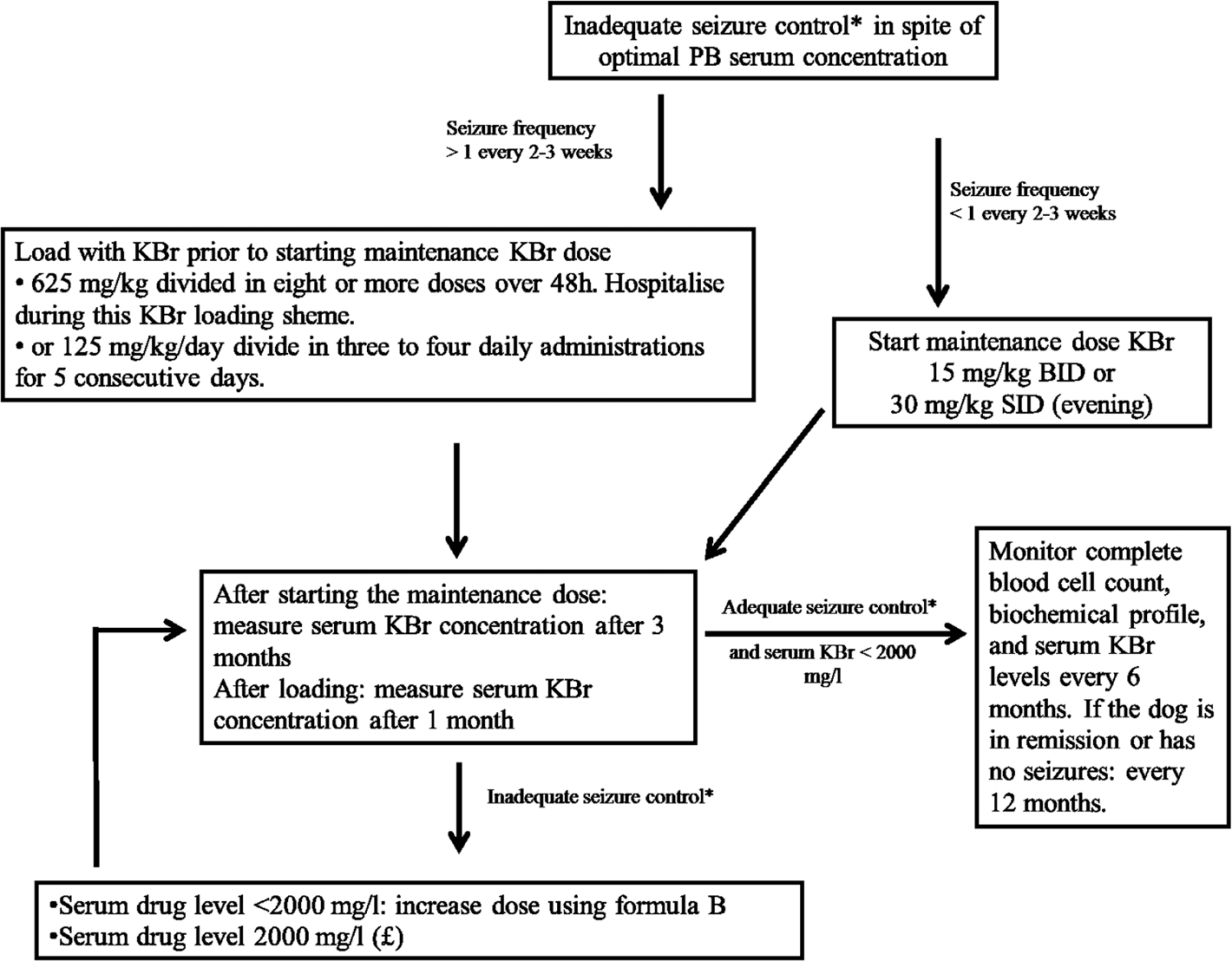
KBr adjunct treatment flow diagram for decision making during seizure management in an otherwise healthy dog. *Criteria for (in)adequate seizure control with regard to efficacy and tolerability (see Consensus proposal: Outcome of therapeutic interventions in canine and feline epilepsy [94]). 1. Treatment efficacious: a: Achievement of complete treatment success (i.e. seizure freedom or extension of the interseizure interval to three times the longest pretreatment interseizure interval and for a minimum of three months (ideally > 1 year), b: Achievement of partial treatment success (i.e. a reduction in seizure frequency including information on seizure incidence (usually at least 50 % or more reduction defines a drug responder), a reduction in seizure severity, or a reduction in frequency of seizure clusters and/or status epilepticus). 2. Treatment not tolerated i.e. appearance of severe adverse effects necessitating discontinuation of the AED

The recommended oral starting dose of KBr is 15 mg/kg BID when used as an add-on drug. An oral dose of 20 mg/kg BID is advised when used as a monotherapy. Because of the long elimination half-life, KBr can be administered once daily (preferably in the evening), however, twice daily dosing as well as administration with food can help to prevent gastrointestinal mucosa irritation [123]. Twice daily dosing is also recommended if excessive sedation is present. Therapeutic ranges have been reported as approximately 1000 mg/l to 2000 mg/l when administered in conjunction with PB and 2000mg/l to 3000mg/l when administered alone [126]. Br has a long half-life, consequently, reaching a steady-state serum concentration may require several months (approximately 3 months). Due to this long half-life, timing of blood sample collection relative to oral administration is not critical [123].

Baseline complete blood cell count, biochemical profile (including cholesterol and triglycerides) should be performed before starting KBr treatment and periodically every 6 months during treatment. Serum KBr concentrations should be monitored 3 months after treatment initiation (or dose change). In the long term, in dogs with adequate seizure control, serum KBr concentrations should be monitored every 6 months. If the dog is in remission or has no seizures, a periodical control every 12 months is advised.

A loading dose may be recommended to achieve steady-state therapeutic concentrations more rapidly (e.g. in dogs with frequent or severe seizures, or when PB must be rapidly discontinued because of life-threatening adverse effects). Different protocols have been reported. Oral loading can be performed by administering KBr at a dose of 625 mg/kg given over 48h and divided into eight or more doses. A more gradual loading can be accomplished giving 125 mg/kg/day divided in three to four daily administrations for 5 consecutive days. Daily phone contact with the owners is advised. Loading can be associated with adverse effects (e.g. nausea, vomiting, diarrhoea, sedation, ataxia and pelvic limb weakness, polydipsia, polyuria and polyphagia) and the dog should be hospitalized if loading takes place over 48h (7,85). It is advised to stop loading when serious adverse effects occur. Consider that dogs in which KBr is used as adjunct AED to PB may be more prone to adverse effects. In these cases, a PB dose decrease of 25 % may be needed. Serum KBr levels should be monitored 1 month after loading.

Dose increases can be calculated according to the following formula

Formula B:

For concomitant PB and KBr treatment, the new maintenance dose can be calculated as follows:

$$ \left(2000\ \mathrm{mg}/\mathrm{l}\ \hbox{-}\ \mathrm{actual}\ \mathrm{serum}\ \mathrm{K}\mathrm{B}\mathrm{r}\ \mathrm{steady}\hbox{-} \mathrm{state}\ \mathrm{concentration}\right) \times 0.02 = \mathrm{mg}/\mathrm{kg}/\mathrm{day}\ \mathrm{added}\ \mathrm{t}\mathrm{o}\ \mathrm{existing}\ \mathrm{dose} $$

Formula C:

In case of monotherapy KBr, the new maintenance dose can be calculated as follows:

$$ \left(2500\ \mathrm{mg}/\mathrm{l} - \mathrm{actual}\ \mathrm{serum}\ \mathrm{K}\mathrm{B}\mathrm{r}\ \mathrm{steady}-\mathrm{state}\ \mathrm{concentration}\right) \times 0.02 = \mathrm{mg}/\mathrm{kg}/\mathrm{day}\ \mathrm{added}\ \mathrm{t}\mathrm{o}\ \mathrm{existing}\ \mathrm{dose} $$

Only PB and imepitoin are approved as first-line treatment of canine epilepsy in the EU. In most EU countries, KBr is only approved as add-on treatment in dogs resistant to first-line treatments. None of the drugs discussed in the following section are approved for treatment of dogs with epilepsy, thus, according to EU drug laws, these drugs can only be used as adjunctive treatment if monotherapy or polytherapy with the approved treatments have failed. Furthermore, except for levetiracetam, none of the AEDs discussed in the following section have been evaluated in randomized controlled trials in epileptic dogs, so that the evidence for their efficacy is very limited [17].

### Levetiracetam

So far, three studies evaluated the efficacy of levetiracetam as an adjunct to other AEDs [79, 114, 127]. In all these studies, the majority of the dogs were treated successfully by oral levetiracetam as adjunct AED. The use of oral levetiracetam was evaluated in an open-label study and a response rate of 57 % was reported in dogs with drug resistant epilepsy [127]. In a recent randomized placebo-controlled study by Muñana et al. (2012) [79], the use of levetiracetam was evaluated in dogs with drug resistant epilepsy. A significant decrease in seizure frequency was reported compared with baseline, however, no difference was detected in seizure frequency when levetiractam was compared with placebo. However, the divergence in group size and the small sample size (due to the high dropout rate) may have contributed to this result. Nevertheless, a trend towards a decrease in seizure frequency and increase in responder rate during levetiracetam administration compared to placebo warrants further evaluation in a larger scale study. According to the study of Charalambous et al., (2014) [17], there is a fair evidence for recommending the use of levetiracetam as an adjunct AED. Recently, a retrospective study provided further evidence that administering levetiracetam as an adjunct AED is well tolerated, and suppresses epileptic seizures significantly in dogs with idiopathic epilepsy [83]. The authors also confirmed that if seizure frequency increases, an extra AED may be beneficial and they added the possibility of administering levetiracetam as pulse treatment for cluster seizures.

Levetiracetam possesses a favourable pharmacokinetic profile in dogs with respect to its use as an add-on AED. It has rapid and complete absorption after oral administration, minimal protein binding, minimal hepatic metabolism and is excreted mainly unchanged via the kidneys. In humans and dogs, renal clearance of levetiracetam is progressively reduced in patients with increasing severity of renal dysfunction [85], thus, dosage reduction should be considered in patients with impaired renal function. As levetiracetam has minimal hepatic metabolism [85], this drug represents a useful therapeutic option in animals with known or suspected hepatic dysfunction. However, its short elimination half-life of 3−6 h necessitates frequent administration. The recommended oral maintenance dose of levetiracetam in dogs is 20 mg/kg TID-QID. The same dose can be administered parenterally in dogs (SC, IM, IV) when oral administration is not possible [86]. In a previous study [127] it was shown that some dogs develop a tolerance to levetiracetam when used chronically. This phenomenon, the ‘honeymoon effect’, has been documented for other AEDs, e.g. zonisamide and levetiracetam in dogs with epilepsy [127, 129]. Therefore, the introduction of the pulse treatment protocol (an initial dose of 60 mg/kg orally or parenterally after a seizure occurs or pre-ictal signs are recognized by the owner, followed by 20 mg/kg TID until seizures do not occur for 48h) was developed, in order to start treatment only in case of cluster seizures when therapeutic levetiracetam concentrations need to be reached rapidly. The results in the recent study by Packer et al., 2015 [83] supports this clinical approach. Pulse treatment was, however, associated with more side effects compared to maintenance levetiracetam therapy [83]. Levetiracetam is well tolerated and generally safe in dogs. Except for mild sedation, ataxia, decreased appetite and vomiting adverse effects are very rarely described in dogs [79, 127] (Table 2). Levetiracetam has also a different mode of action compared to other AEDs and therefore may be advantageous when polytherapy is instituted. It selectively binds to a presynaptic protein (SVA2), whereby it seems to modulate the release of neurotransmitters [86]. As, in dogs there is no information available regarding a therapeutic range [79], the human target range of 12−46 μg/l can be used as guidance regarding effective concentrations.

**Table 2:** Most common reported adverse effects seen in dogs treated with levetiracetam, zonisamide, felbamate, topiramate, gabapentin, and pregabalin (rarely reported and/or idiosyncratic adverse effects are indicated in grey

AED	Adverse effects in dogs
Levetiracetam	Sedation
	Ataxia
	Decreased appetite or anorexia
	Vomiting
	Behavioural changes
Zonisamide	Sedation
	Ataxia
	Vomiting
	Inappetence
	
	
Felbamate	Keratoconjunctivitis sicca
	Thrombocytopenia
	Lymphopenia and leucopenia
Topiramate	Sedation
	Ataxia
	Weight loss
Gabapentin	Sedation
	Ataxia
Pregabalin	Sedation
	Ataxia
	Weakness

Studies in humans have shown that concomitant administration of AEDs that induce cytochrome P450 metabolism such as PB, can alter the disposition of levetiracetam [19]. Recently, it has been demonstrated that PB administration significantly alters the pharmacokinetics of levetiracetam in normal dogs [73]. Thus, levetiracetam oral dose may need to be increased or dosing time interval may need to be shortened when concurrently administered with PB [73]. Also in dogs with epilepsy, concurrent administration of PB alone or in combination with KBr increases levetiracetam clearance compared to concurrent administration of KBr alone [78]. Thus, dosage increases might be indicated when utilizing levetiracetam as add-on treatment with PB in dogs [78], preferably guided by levetiracetam serum concentration measurement.

### Zonisamide

There are few reports on the use of zonisamide in dogs, despite it being licensed for treatment of canine epilepsy in Japan. One report evaluated the efficacy of oral zonisamide as a monotherapy [18]. Two studies have been described evaluating zonisamide as an add-on treatment in dogs with drug resistant epilepsy [28, 129]. Based on the results of these studies, Charalambous et al. (2014) [17] concluded that, at present, there is insufficient evidence to recommend the use of zonisamide either as a monotherapy or as an adjunct AED in dogs. Larger studies are required to evaluate zonisamide as a monotherapy or as an adjunctive AED in dogs. Adverse effects in dogs include sedation, vomiting, ataxia, and loss of appetite [18, 28, 129] (Table 2). Additionally, recently hepatotoxicity has been described in 2 dogs receiving zonisamide monotherapy which is believed to be an idiosyncratic reaction to the drug [69, 104] (Table 2). Renal tubular acidosis has also been described in a dog receiving zonisamide monotherapy [20] (Table 2). Thus, zonisamide should be used with caution in dogs with renal or hepatic impairment. Both, hepatic and renal failures have been described in humans receiving zonisamide as well. Currently, zonisamide is not available in every country and when available, it can be very expensive.

Zonisamide is a sulphonamide-based anticonvulsant approved for use in humans. The exact mechanism of action is unknown, however, blockage of calcium channels, enhancement of GABA release, inhibition of glutamate release, and inhibition of voltage-gated sodium channels might contribute to its anticonvulsant properties [61]. In dogs, zonisamide is well-absorbed after oral administration, has a relatively long elimination half-life (approximately 15h), and has low protein binding so that drug interactions are minimized. The drug mainly undergoes hepatic metabolism via the cytochrome P450 system before excretion by the kidneys [11].

The recommended oral starting dose of zonisamide in dogs is 3−7 mg/kg BID and 7−10 mg/kg BID in dogs co-administered hepatic microsomal enzymes inducers such as PB [11, 28]. Serum concentrations of zonisamide should be measured minimally 1 week after treatment initiation or dosage adjustment to allow steady state concentrations be reached. Care should be taken to avoid haemolysis, as falsely elevated serum zonisamide concentrations from lysed red blood cells may occur. The human target range of 10−40 mg/l can be used as guidance regarding effective concentrations. [28]. Baseline complete blood cell count and biochemical profile should be performed before starting zonisamide treatment and periodically every 6 months during treatment.

### Felbamate

One veterinary study evaluated the efficacy of felbamate as an adjunct to PB in 6 dogs with focal idiopathic epilepsy [100]. According to Charalambous et al. (2014) [17], the study demonstrated overall moderate/high risk of bias. On this basis it was concluded that there is currently insufficient evidence to recommending the use of felbamate as an add-on AED. Felbamate should be reserved for dogs refractory to the other more thoroughly investigated and safer AEDs in this species and as such this is a 4^th^ or 5^th^ line option. In the clinical study by Ruehlmann et al., (2001) [100] adverse effects noted included keratoconjunctivitis sicca and mild blood dyscrasias (Table 2).

Felbamate is a dicarbamate AED released for use in humans in 1993 for the control of focal seizures. Its mechanism of action is multiple such as inhibition of glycine-enhanced NMDA-induced intracellular calcium currents [134], blockade of voltage-gated sodium channels and inhibition of voltage –gated calcium currents [133].

In 1993, felbamate was marketed as a safe AED, which lacked demonstrable toxic side effects and did not require laboratory monitoring in humans. However, within a year of its release it became evident that felbamate was associated with an unacceptable incidence of life-threatening side effects [12], such as anorexia, weight loss, vomiting, headache, irritability. Moreover, aplastic anemia and fatal hepatotoxicity were also described [55, 134].

Pharmacokinetic interactions between felbamate and other AEDs have been well described. E.g. felbamate raises concurrent PB serum levels in a dose-dependent manner [12], and the elimination of felbamate was noted to be strikingly reduced when given with gabapentin [50]. Felbamate is mainly metabolized by the liver [88] and should therefore not be used in dogs with pre-existing hepatic disease. Felbamate has an elimination half-life of 5−7h.

The recommended oral starting dose in dogs is 20 mg/kg TID, increasing to 400−600mg/day every 1−2 weeks [1]. Haematologic evaluations and biochemistry panels (esp. liver enzyme concentrations) should be performed before felbamate therapy is initiated and during therapy. This is especially important in animals receiving concurrent PB. In humans, the signs of aplastic anaemia and liver failure are usually seen during the first 6−12 months of therapy. In dogs, a minimum of monthly blood tests should be performed for this period of time, following-up every 6−12 months after this. Currently, felbamate is not available in every country.

### Topiramate

In 2013, one sudy evaluated the efficacy of topiramate as an adjunct to PB, KBr, and levetiracetam in 10 dogs [57]. The dose was titrated (2−10 mg/kg) two to three times daily. Sedation, ataxia and weight loss were the most common adverse effects in dogs (Table 2). According to Charalambous et al. (2014) [17], the study demontsrated an overall moderate/high risk of bias. Thus, there is currently insufficient evidence to recommend the use of topiramate as an adjunct AED [17].

In humans, topiramate has served both as a monotherapy and adjunctive therapy to treat focal and generalised seizures [29, 71]. It is a sulphamate-substituted monosaccharide that acts on multiple signalling mechanisms enhancing GABA-ergic activity and inhibiting voltage-sensitive sodium and calcium channels, kainate-evoked currents and carbonic anhydrase isoenzymes [118, 139].

From the available human data, topiramate is not metabolized extensively once absorbed, with 70−80 % of an administered dose eliminated unchanged in the urine [65]. Topiramate has an elimination half-life of 2−4h. Clearance of topiramate is reduced in patients with renal impairment, necessitating dosage adjustments [37]. In dogs, topiramate is not extensively metabolized and is primarily eliminated unchanged in the urine. However, biliary excretion is present following topiramate administration in dogs [15]. The drug has a relatively low potential for clinically relevant interactions with other medications [8, 53]. The most commonly observed adverse effects in humans are somnolence, dizziness, ataxia, vertigo and speech disorders [110]. No adverse reactions were reported in healthy Beagle dogs administered 10−150 mg/kg daily oral doses for 15 days [116].

### Gabapentin

Two prospective studies evaluated the efficacy of oral gabapentin as an adjunct to other AEDs, giving a combined sample size of 28 dogs [44, 89]. According to Charalambous et al. (2014) [17], one study demonstrated an overall moderate/high risk of bias and the other one demonstrated an overall high risk of bias. None of the studies demonstrated an increased likelihood that the majority of the dogs were treated successfully by oral administration of gabapentin. Accordingly, there is currently overall insufficient evidence for recommending the use of gabapentin as an adjunct AED [17]. If used, the recommended oral dosage of gabapentin in dogs is 10 to 20 mg/kg TID, although dose reduction may be necessary in patients with reduced renal function [9]. Sedation and ataxia were the most common side effects reported in dogs [44, 89] (Table 2).

Gabapentin has been approved in people in Europe and by the US Food and Drug Administration (FDA) since 1993 for adjunctive treatment of focal seizures with or without secondary generalisation and for the treatment of post-herpetic neuralgia [9]. Its precise mechanism of action is unclear, but is believed that much of its anticonvulsant effect is because of its binding to a specific modulatory protein of voltage-gated calcium channels, which results in decreased release of excitatory neurotransmitters [112]. In humans, gabapentin is entirely excreted by the kidneys. In dogs, renal excretion occurs after a partial hepatic metabolism. The elimination half-life is 3−4h.

Although information in veterinary medicine is limited, pharmacokinetic interactions of gabapentin are unlikely to occur as the drug has negligible protein binding and does not induce hepatic cytochrome P450 family enzymes [95]. In humans, the elimination of felbamate was noted to be significantly reduced when given with gabapentin [50]. The most common adverse effects in humans include dizziness, somnolence and fatigue [9]. These effects seem to be dose-dependent and resolve within the first few weeks of treatment. No serious idiosyncratic reactions or organ toxicities have been identified in humans or animals [60].

### Pregabalin

There is limited data on the use of pregabalin in dogs. In a study by Dewey et al., (2009), the efficacy of oral pregabalin as an adjunct to PB and KBr was evaluated in 9 dogs [27]. According to Charalambous et al. (2014) [17], this study demonstrated an overall moderate/high risk of bias. Consequently, there is currently insufficient evidence to recommend the use of pregabalin as an adjunct AED [17]. If used, the recommended oral dose in dogs is 3−4 mg/kg BID-TID. The most common adverse effects (Table 2) in the study of Dewey et al., (2009) included sedation, ataxia and weakness, and to minimize these, treatment could be initiated at a dose of 2 mg/kg two to three times daily and escalated by 1 mg/kg each week until the final dose is achieved [27]. As pregabalin clearance is highly correlated with renal function, dose reduction is necessary in patients with reduced renal function [5, 9].

Pregabalin is a GABA analogue that is structurally similar to gabapentin. Pregabalin was approved in 2004 for the treatment of adults with peripheral neuropathic pain and as adjunctive treatment for adults with focal seizures with or without secondary generalization. Pregabalin is more potent than gabapentin owing to a greater affinity for its receptor [112]. Pharmacokinetic studies have been performed in dogs, with a reported elimination half-life of approximately 7 h [103]. In humans, pregabalin does not bind to plasma proteins and is excreted virtually unchanged by the kidneys [9]. Pregabalin does not undergo hepatic metabolism and does not induce or inhibit hepatic enzymes such as the cytochrome P450 system [5]. No clinically relevant pharmacokinetic drug interactions have been identified in humans to date. The most commonly reported adverse effects in humans are dose-related and include dizziness, somnolence and ataxia [9].

#### Discontinuation of AEDs

Two main reasons for discontinuation of an AED are remission of seizures or life-threatening adverse effects. Generally, treatment for idiopathic epilepsy involves lifelong AED administration. However, remission has been reported in dogs. Remission rates between 15−30 % have been described in hospital based populations [6, 7, 47, 49]. In a study by Packer et al. (2014) 14 % of dogs were in remission on PB [84]. When ≥50 % reduction in seizure frequency was used as the outcome measure, success rates were markedly higher with 64,5 % of dogs achieving this level of seizure reduction. Several factors were associated with an increased likelihood of achieving remission, namely: being female, neutered, no previous experience of cluster seizures and an older age at onset of seizures. The same four factors were associated with an increased likelihood of achieving a ≥50 % reduction in seizure frequency [84]. The breed least likely to go into remission or have an ≥50 % reduction in seizure frequency was the Border Collie (0 and 40 %, respectively), the German Shepherd (11 and 35 %, respectively) and Staffordshire Bull Terrier (0 and 57 %, respectively) [84]. In a study by Hülsmeyer et al. (2010) the remission rate was 18 % in Border Collies independent of disease severity [49]. The decision to gradually taper the dose of an AED should be taken on a case-by-case basis, but seizure freedom of at least 1−2 years is advised. In people with prolonged seizure remission (generally 2 or more years), the decision to discontinue AED treatment is done on an individual basis considering relative risks and benefits. Individuals with the highest probability of remaining seizure-free are those who had no structural brain lesion, a short duration of epilepsy, few seizures before pharmacological control, and AED monotherapy [81, 109]. In dogs, however, little information on risk factors associated with seizure relapse exist, thus the pet owner must be aware that seizures may recur anytime during AED dose reduction of after discontinuation. To prevent withdrawal seizures or status epilepticus it is advised to decrease the dose with 20 % or less on a monthly basis.

In case of life-threatening adverse effects, instant cessation of AED administration under 24h observation is necessary. In these cases, loading with an alternative AED should be initiated promptly in order to achieve target serum concentrations before serum PB concentration decreases. Loading with KBr (see section on KBr) or levetiracetam (see section on levetiracetam) is possible. If hepatic function is normal, starting imepitoin or zonisamide at the recommended oral starting dose may be another alternative.

#### Pet owner education

In order to promote a successful management of an epileptic pet, owners need to be educated thoroughly on [23, 32, 91]:

- The disease of their pet and the influence on their daily life (considerations regarding e.g. leaving the dog alone, what to do if travelling and leaving the dog in a kennel, fears of behavioural comorbidities, …)
- The need for AED therapy and the understanding that this often is a lifetime commitment
- The aim of AED therapy
- The importance of regular administration of AEDs
- The fact that dose adjustments should only be made after consulting a veterinarian
- Potential adverse effects of AED therapy
- The importance of maintaining a detailed seizure diary
- The importance of regular check-ups to monitor AED blood concentrations as well as haematology/serum biochemistry where appropriate
- The need for treatment modulation to achieve optimal seizure control
- The possibility of occurrence of status epilepticus and cluster seizures and the administration of additional AEDs at home
- Costs involved
- The fact that drug interactions might occur when combined with other AEDs or non-AEDs
- The understanding that abrupt drug withdrawal might be detrimental
- The fact that diet (e.g salt content), diarrhoea and vomiting may affect the absorption of AEDs. It should be advised to keep the diet constant or to make changes gradually and seek veterinary advice if gastrointestinal signs occur.

## Abbreviations

AED: Antiepileptic drug
PB: Phenobarbital
KBr: Potassium bromide
Br: Bromide
IM: Intramuscular
IV: Intravenous
PO: Orally
SC: Subcutaneously
SID: Once daily
BID: Twice daily
TID: Three times daily
QID: Four times daily
